# The role of miR-29c/B7-H3/Th17 axis in children with *Mycoplasma pneumoniae* pneumonia

**DOI:** 10.1186/s13052-019-0655-5

**Published:** 2019-05-14

**Authors:** Qing-ling Li, Yin-yin Wu, Hui-ming Sun, Wen-jing Gu, Xin-xing Zhang, Mei-juan Wang, Yong-dong Yan, Chuang-li Hao, Wei Ji, Zheng-rong Chen

**Affiliations:** Department of Respiratory Disease, Children’s Hospital of Soochow University, Jingde Road NO.303, Jiangsu Province, Suzhou, 215003 China

**Keywords:** *Mycoplasma pneumoniae* pneumonia, *Children*, *MircoRNA*, *B7-H3*, *The cell differentiation*

## Abstract

**Background:**

*Mycoplasma pneumoniae* (*M. pneumoniae*) is one of the most common causes of community-acquired pneumonia in children. Recent studies demonstrated that the incidence of severe or fatal *M. pneumoniae* was gradually increasing, which may be related to the excessive inflammation. However, the exact pathogenesis of excessive inflammation in *Mycoplasma pneumoniae* pneumonia(MPP) is still unclear. This study aimed to reveal the role of miR-29c/B7-H3/Th17 axis in children with MPP.

**Methods:**

Children hospitalized in Respiratory Department during Jan. 2014 to Dec. 2015 were enrolled. All children enrolled was confirmed with MP infection using real-time PCR and ELISA. Children were excluded if they were co-infected with other pathogens. A total of 52 children with MPP and 26 controls were enrolled. miR-29c expression in monocytes of children with MPP was determined by real-time PCR and soluble B7-H3 (sB7-H3) and IL-17 were determined by ELISA, and explore their clinical significance. miR-29c overexpression and silencing technology and luciferase reporter assay were performed to confirm whether B7-H3 is the direct target of miR-29c. The levels of transcription factor ROR-γt in CD4+ T cells and cytokine IL-17A in supernatant were detected after stimulated by different concentrations of B7-H3 fusion protein in vitro.

**Results:**

Of all 52 children with MPP, the mean age of the children were 77 ± 33 months, and 23 cases were male accounting for 44.2%. Nineteen cases had pleural effusion accounting for 36.5%. Children with MPP had significantly lower level of miR-29c and higher level of sB7-H3 and IL-17 compared to controls (both *P* < 0.05). The level of miR-29c significantly increased during convalescent phase compared to that of acute phase while sB7-H3 and IL-17 significantly decreased during convalescent phase (both P < 0.05). There was a positive correlation between the level of sB7-H3 and IL-17 in children with MPP during acute-stage (r = 0.361,*P* = 0.009). Children with MPP combined with pleural effusion had significantly higher level of sB7-H3 compared to those without pleural effusion (9952.3 ± 3065.3 vs. 7449.7 ± 2231.5, pg/ml), and the levels of sB7-H3 was positively correlated with the number of days of fever. The level of miR-29c was negatively correlated with *M. pneumoniae* specific IgG, IgM level. High concentrations of B7-H3(15μg/ml) could enhance ROR-γt expression and increase IL-17A. Functional studies based on luciferase reporter assay and immunofluorescence staining suggested that B7-H3 is the direct target of miR-29c, and miR-29c silencing or overexpression could up- or down-regulate the expression of B7-H3 in THP-1 cells.

**Conclusions:**

The axis of miR-29c/B7-H3/Th17 plays a vital role in children with MPP through excessive inflammation. miR-29c and B7-H3 may be the new target for the prevention and treatment of MPP, and may be the novel and potential biomarkers for the assessment of prognosis.

## Introduction

*Mycoplasma pneumoniae* (*M. pneumoniae*) is one of the most common causes of respiratory tract infection in children, which can lead to upper and lower respiratory tract infection, especially in Community-acquired pneumonia (CAP). According to statistics, MP causes up to 40% or more of CAP in children and as many as 18% of cases requiring hospitalization [1]. Mycoplasma pneumoniae pneumonia (MPP) was usually a mild and self-limiting disease, and the therapy of macrolides antibiotics for MPP were always effective. It was reported that the incidence of severe or fatal MPP was gradually increasing [2] .The clinical features of severe MPP (SMPP) include large amount of pleural effusion, acute respiratory distress syndrome (ARDS), pulmonary fibrosis, obstructive bronchiolitis and even life threatening. Meanwhile, the lingering effects of SMPP include bronchiectasis, atelectasis, obstructive bronchiolitis etc.

However, the etiology and pathogenesis of SMPP remains unclear, it was reported that excessive host immune reactions played a part in it, and lymphocytes were mainly involved in this form, such a the excessive immune reactions caused by Th1 and Th17 [3, 4] Th cells activation and differentiation is associated with costimulatory molecules expressed on antigen presenting cell including B7 family molecules. Our previous study showed that children with MPP had significantly higher level of sB7-H3, which was positively correlated with the level of proinflammatory TNF-α [5]. Chapoval AI reported that B7-H3 could specifically enhance the production of Th1-type cytokines interferon gamma (IFN-γ) [6]. Taken together, these studies indicated that co-stimulatory molecule B7-H3 played an important role in development of MPP through regulating of Th differentiation.

How dose the gene expression of B7-H3 be regulated? Previous studies indicated that MicroRNAs (miRNAs) could regulate gene expression transcriptionally or post-transcriptionally, and play roles in the development excessive inflammation due to the immune reaction [7]. Recently, it has been found that B7-H3 expression levels was high in melanoma cells and overexpression of miR-29c could reduce the expression of B7-H3. MiR-29c expression was shown to inversely regulate B7-H3 expression in melanoma cells [8].

Thus,we hypothesized that the axis of miR-29c/B7-H3/Th17 plays a vital role in the development of MPP. Our study aimed to explore the role and clinical significance of mir-29c and B7-H3 in children with MPP, and the mechanism of miR-29c and B7-H3 on Th17 differentiation, and to provide new clues for prevention and treatment of MPP.

## Materials and methods

### Object of study

Children hospitalized in Respiratory Department of Children’s Hospital of Soochow University during Jan. 2014 to Dec. 2015 were enrolled. The Children were defined as MPP with the following criteria: 1) M.pneumoniae DNA was detected in nasopharyngeal aspirates by real-time polymerase chain reaction (PCR) and specific IgM and IgG antibodies against M. pneumoniae in paired sera by enzyme-linked immunosorbent assays (ELISA). 2) Patients were excluded if they were were co-infected with other pathogens. A total of 52 children with MPP were enrolled at last. The male-to-female ratio was 0.79 to 1. The mean age of the cases with MPP were 77 ± 33 months. Demographic and clinical information were collected in all patients including age, gender, duration, duration of fever, length of hospitalization, complications, and medication. Imaging results were conducted using chest radiography, chest CT, and thoracic ultrasound. Laboratory specimens were obtained including peripheral blood, and nasopharyngeal aspirates. The following laboratory tests were conducted including blood routine, C-reactive-protein (CRP), biochemicalfunction, humoral-immunity, and lymphocyte subsets. A total of 26 controls with age matched were selected from children with elective surgery including inguinal hernia, multiple fingers deformity, and fracture fixation after internal fixation from surgery wards of Children’s Hospital of Soochow University. All the controls enrolled in this study without history of infections, drugs allergy, family or personal allergy within 4 weeks. There was no significant difference in the age of children between MPP group and control group. This study was approved by the Institutional Human Ethical Committee of Children’s Hospital of Soochow University. A written consent was obtained from all the guardians who participated in this study.

## Study method

### Peripheral blood and nasopharyngeal secretions samples

Venous blood samples of all these children were collected within 24 h of admission,and immediately sent to the laboratory. The specimen was centrifugated at 3000 r/min for 5 min, then collected supernantant and separate them with EP tubes. The second peripheral blood samples were collected before discharge. All specimens were stored at − 70 °C for subsequent assay. As for nasopharyngeal aspirates, the samples of all these children were collected within 24 h of admission.

### Quantitative ELISA specific M. pneumoniae IgG and IgM

Specific IgM and IgG antibodies against *M. pneumoniae* were detected in serum samples of patients in the acute phase of *M. pneumoniae* pneumonia (on admission) and in the convalescent phase (on discharge), respectively, using a commercial ELISA kit (Serion ELISA classic MP IgG/IgM, Institute Virion/Serion, Würzburg, Germany) according to the manufacturer’s instructions. The test cut-off value was 0.5 × mean optical density (OD) of the kit control serum, as indicated in the insert. A positive IgG reaction was defined as > 24 RU/mL. A significant rise in IgG titre was considered to be a doubling of the OD value above the cut-off, or a sero-conversion in which the primary serum was antibody negative and the second serum had an OD at least twice the cut-off corresponding to a threefold rise in RU/mL titre. A positive IgM antibody reaction was defined as > 1.1 S/CO.

### Real-time PCR for M. pneumoniae detection

Nasopharyngeal aspirates were obtained within 24 h of admission. The samples were shaked, centrifuged, and then removed liquid supernatant, added lysis buffer and were stored at − 80 °C. A quantitative diagnostic kit (DaAn Gene Co., Ltd. Guangzhou, China) for *M. pneumoniae* DNA was used to measure the load of *M. pneumoniae*. The method is based on TaqMan PCR technology, and the target is 16S rRNA gene specific for *M. pneumoniae* genome. Briefly, 1 mL of nasopharyngeal aspirates diluted by 4% NaOH was centrifuged at 12,000 rpm for 5 min. The sediment was collected, washed twice with 0.9% NaCl, blended with 50 μL of DNA extraction solution, incubated at 100 °C for 10 min, and centrifuged at 12,000 rpm for 5 min. Real-time PCR was performed on the resulting supernatant of 2 μL with 43 μL of PCR mix (supplied with the kits) using the DA 7600 real-time PCR system (Applied Biosystems, CA, USA) as follows: 93 °C for 2 min, 10 cycles of 93 °C for 45 s and 55 °C for 60 s, followed by 30 cycles of 93 °C for 30 s and 55 °C for 45 s.

### Multiple pathogen detection

seven common viruses in the respiratory tract were detected using direct immunofluorescence assay including respiratory syncytial virus, adenovirus influenza virus types A and B, parainfluenza virus types 1–3. Detection kits were purchased from Chemieon company, USA. All procedures were conducted according to the manufacturer^,^s instructions. Human metapneumovirus were detected using RT-PCR, and human bocavirus were detected using fluorogenic quantitative PCR as described previously [9, 26].

### Examination of soluble B7-H3 and IL-17 in plasma

The levels of soluble B7-H3 and IL-17 in peripheral blood were detected by ELISA. The procedure is according to the manufacture’s instructions. Soluble B7-H3 kits were purchased from Xuguang Technology Co.Ltd. Suzhou.IL-17 ELISA kits were purchased from R&D Systems company, USA.

### Determination of miR-29c in peripheral blood monouclear cells

Isolated peripheral blood mononuclear cells were collected, Cell lysis, organic extraction, miRNA enrichment were performed using the mirVanaTM miRNA Isolation Kit (Ambion company) to extract total miRNA. The procedure is according to the manufacture’s instructions.. Briefly, 10 ng of total RNA was subjected to reverse transcription polymerase chain reaction using the TaqMan MicroRNA Reverse Transcription kit (Applied Biosystems) according to manufacturer’s protocol. The thermocycling conditions were: 30 min at 16 °C, followed by 30 min at 42 °C, 5 min at 85 °C and 5 min at 4 °C. qRT-PCR was performed using TaqMan Universal PCR Master Mix Kit(Applied Biosystems) in a Bio-Rad iQ5 Real-Time PCRSystem and U6 was used as an endogenous control. The reaction was performed in triplicate according to manufacturer’s protocol. The thermocycling conditions were: 50 °C for 2 min, 95 °C for 10 min, and 40 cycles of 15 s at 95 °C, followed by 1 min at 60 °C. After finalization of the qRT-PCR experiments, the average values of the cycle threshold (Ct) of the reactions in triplicate were determined. Data analysis was performed using the 2^-ΔΔ^Ct method.

### B7-H3 regulated the expression of ROR-γt and secretion of IL-17A in CD4+ T cells

CD4^+^ T cells (1 × 10^5^ cells/ml) were isolated from peripheral blood of healthy human (*n* = 3, donate from Red Cross, Suzhou, China) using autoMACS columns with CD4^+^ T cell isolation kit (Miltenyi Biotec). After a 24 h culture at 37 °C, 5% CO_2_, proliferated T cells were harvested and seeded into 6-well flat-plate that was pre-coated with anti-CD3 mAb (50 ng/ml) and anti-CD28 mAb (500 ng/L) purchased from Bright Scistar Biotech, Suzhou, China. Then a different dose of B7-H3 (0 μg/ml, 0.6 μg/ml, 3 μg/ml and 15 μg/ml) was also added into the wells. After another 24 h, cell-free supernatants were collected to measure cytokines of IL-17 while cells were collected to measure the relative expression of mRNA of ROR-γt.

### Transfection of miR-29c and B7-H3 detection by immunofluorescence staining

THP-1 cells (ATCC, Manassas, VA, USA) were seeded at 1 × 10^5^ cells/60 mm dishes and then transfected with 100 nM pLenti-miR-29c, anti-miR-29c or empty vector using the Jetprime™ Transfection Reagent (VWR International, Radnor, PA). After transfection (24 h), cells were treated with 1 mg/ml Pronase E (E. Merck, Darmstadt, Germany) for 30 min at 37 °C to strip off B7-H3 protein already on the cell surface, and another 48 h later newly expressed B7-H3 protein level were measured by anti-B7-H3 mAb (8H9) immunofluorescence staining. Slides were imaged using a digital slide scanner and grey levels of slides were obtained using Image-Pro Plus software.

### 3′-UTR reporter constructs and luciferase assays

The B7-H3 3′-UTR-WT and mutatant was purchased from NOVOBIO (Shanghai, China). Oligonucleotides corresponding to the miR-29c binding site in the B7-H3 3’UTR or a single-base mutant were synthesized and inserted into the XbaI site immediately downstream from the stop codon of firefly luciferase of the pGL3-control vector (Novobio Co. Ltd., Shanghai, China). Human monocytic cell line THP-1 was obtained from American Type Culture Collection (ATCC, Manassas, VA, USA). THP-1 cells were co-transfected in 24-well plates using Lipofectamine 2000 reagent (Invitrogen) according to the manufacturer’s protocol, with 50 ng of the firefly luciferase reporter, 1 ng of the renilla luciferase reporter (Promega) as transfection control, and 100 nM pLenti-miR-29c (Novobio Co. Ltd., Shanghai, China). Firefly and renilla luciferase activities were measured sequentially using dual-luciferase assays (Promega) 24 h after the transfection and evaluated by the BioTek™ Microplate Reader.

### Data analysis

Normal distribution of measurement data were expressed as ($\overline{x}$ ±*SD*), the Student T-test were performed for the comparisons between the two groups. Non-normal distribution of measurement data were expressed as median (Quartile spacing), and Wilcoxon test was performed for the comparisons between the two groups. The Chi-square test or Fisher’s exact test were applied for numeration data. Statistical analysis was performed using SPSS 18.0 software package. A two-sided *p*-value of < 0.05 was considered statistically significant.

## Results

### Demographic data, clinical and laboratory characteristics of children with MPP

Total of 52 children with MPP were enrolled in this study. The demographic data, clinical and laboratory characteristics of children with MPP are shown in Table 1. The mean age of the children were 77 ± 33 months, and 23 were male, accounting for 44.2%, 19 of them had pleural effusion, accounting for 36.5%.

**Table 1:** Demographic and clinical profiles of children with MPP

Parameters	Childen with MPP *n* = 52
Age (mean ± SD, months)	77 ± 33
Male (n, %)	23 (44.2)
Duration of fever, (mean ± SD, d)	7.5 ± 3.2
Length of stay, (mean ± SD, d)	8.9 ± 2.7
White blood cell counts (mean ± SD, × 10^9^/L)	8.6 ± 3.7
Neutrophils (mean ± SD, × 10^9^/L)	63.8 ± 13.3
C-reactive protein (25th–75th percentile, mg/L)	16.1 (4.8–43.5)
L-lactate dehydrogenase (mean ± SD, U/L)	468.4 ± 190.3
Lymphocyte subgroups (mean ± SD, %)	
CD3+	65.7 ± 9.6
CD3 + CD4+	34.2 ± 7.8
CD3 + CD8+	27.0 ± 6.9
CD3-CD19+	20.4 ± 7.3
CD3-CD(16 + 56+)	12.3 ± 7.9
CD19 + CD23+	9.8 ± 4.6
*M. pneumoniae* specific IgM (25th–75th percentile, S/CO)	23.7 (8.6–57.7)
*M. pneumoniae* specific IgG (mean ± SD, RU/ml)	2.3 ± 1.7

*MPP Mycoplasma pneumoniae* pneumonia, *SD* standard deviation

### Expression of miR-29c, sB7-H3 and IL-17 in children with MPP

**Fig. 1:**
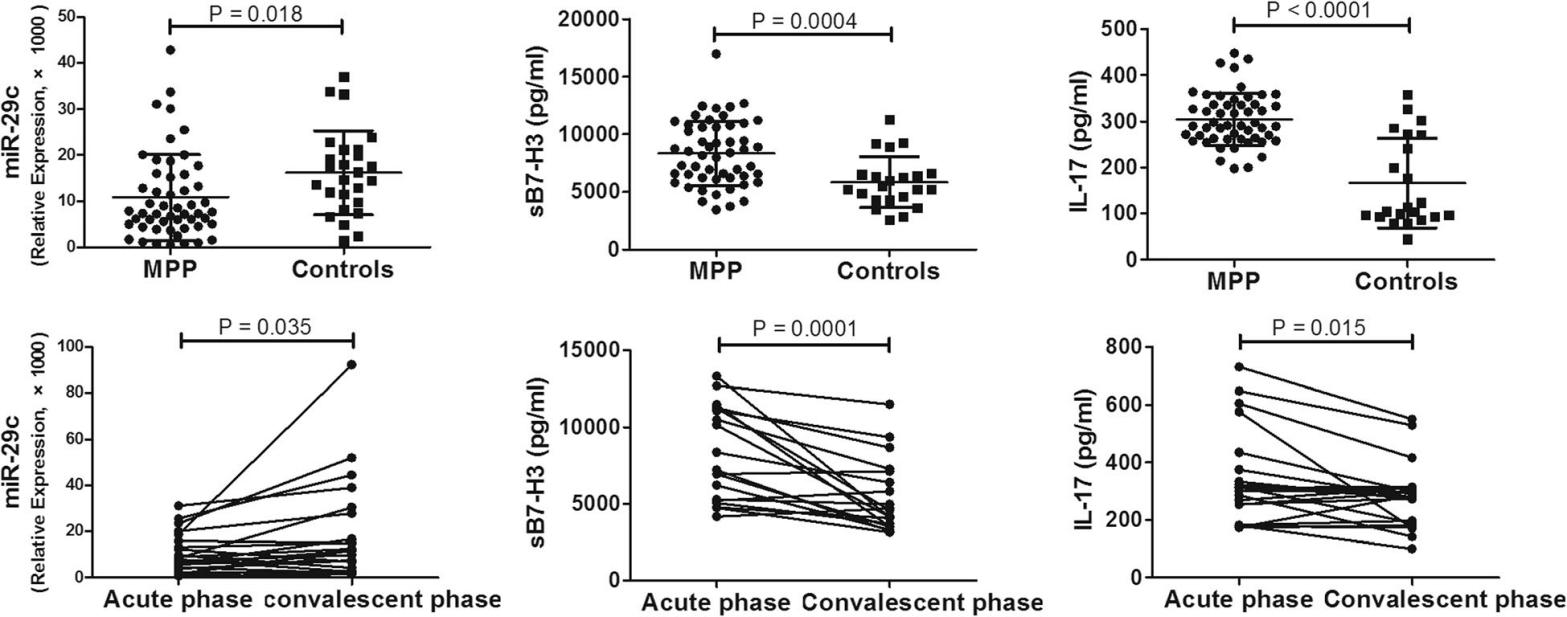
Expression of miR-29c, sB7-H3 and IL-17 in children with MPP. *P* < 0.05 was considered statistically significant

AS shown in Fig. 1, children with MPP had significantly lower level of miR-29c and higher level of sB7-H3 and IL-17 compared to controls (both *P* < 0.05). Peripheral blood were obtained from 22 cases of children with MPP during the convalescent phase (at least 1 week late), and the level of miR-29c, sB7-H3 and IL-17 were detected. It shown that level of miR-29c significantly increased during convalescent phase compared to that of acute phase while sB7-H3 and IL-17 significantly decreased during convalescent phase. The level of sB7-H3 were positively correlated with IL-17 (r = 0.361,*P* = 0.009).

### Comparision of miR-29c, sB7-H3, and IL-17 in MPP cases with and without pleural effusion

**Fig. 2:**
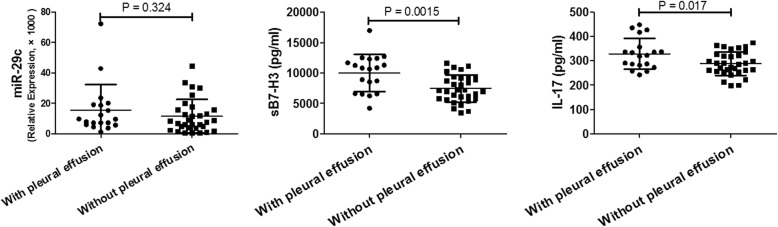
Comparision of miR-29c, sB7-H3, and IL-17 in MPP cases with and without pleural effusion. P < 0.05 was considered statistically significant

Pleural effusion is an indicator and clinical manifestation of severe inflammatory response. Therefore, these inflammatory factors mentioned above are explored in MPP cases with pleural effusion. As shown in Fig. 2, children with MPP combined with pleural effusion had significantly higher level of sB7-H3 compared to to those without pleural effusion (9952.3 ± 3065.3 vs. 7449.7 ± 2231.5, pg/ml,*P* = 0.0015). And there was no significant differences between the two groups in the levels of miR-29c and IL-17.

### Correlation between the levels of miR-29c, sB7-H3, IL-17 and clinical parameters in children with MPP

As shown in Table 2, the level of sB7-H3 was positively correlated with the duration of fever (*P* < 0.05), The level of miR-29c was negatively correlated with the *M. pneumoniae* specific IgG and IgM levelboth (both P<0.05). No significant differences was found between sB7-H3, miR-29c and other clinical parameters..

**Table 2:** Correlation between the levels of miR-29c, sB7-H3, IL-17 and clinical parameters in children with MPP

Parameters	miR-29c		sB7-H3		IL-17	
	r	P	r	P	r	P
Duration of fever	0.025	0.86	0.350	0.011	0.188	0.181
Length of stay	0.095	0.505	0.252	0.072	0.097	0.473
WBC	− 0.095	0.501	0.241	0.086	0.142	0.314
Neutrophils (%)	0.085	0.547	0.153	0.279	0.061	0.666
C-reactive protein	0.127	0.369	0.214	0.128	0.246	0.079
L-lactate dehydrogenase	0.103	0.467	0.272	0.056	0.240	0.093
CD3^+^	0.095	0.505	0.252	0.072	0.197	0.094
CD3^+^CD4^+^	−0.018	0.905	− 0.131	0.375	− 0.100	0.500
CD3^−^CD8^+^	0.065	0.659	−0.191	0.194	−0.050	0.737
CD3^−^CD19^+^	0.068	0.648	−0.212	0.147	−0.008	0.958
CD3^−^CD(16^+^ 56^+^)	0.097	0.511	0.039	0.794	−0.036	0.808
CD19^+^CD23^+^	−0.073	0.624	0.100	0.497	0.132	0.373
*M. pneumoniae* IgG	−0.374	0.007	0.157	0.272	−0.086	0.551
*M. pneumoniae* IgM	−0.377	0.006	−0.008	0.957	−0.130	0.362

*MPP Mycoplasma pneumoniae* pneumonia, *WBC* white blood cell

### Function of B7-H3 on CD4^+^ T cells on expression of ROR-γt and IL-17A

**Fig. 3:**
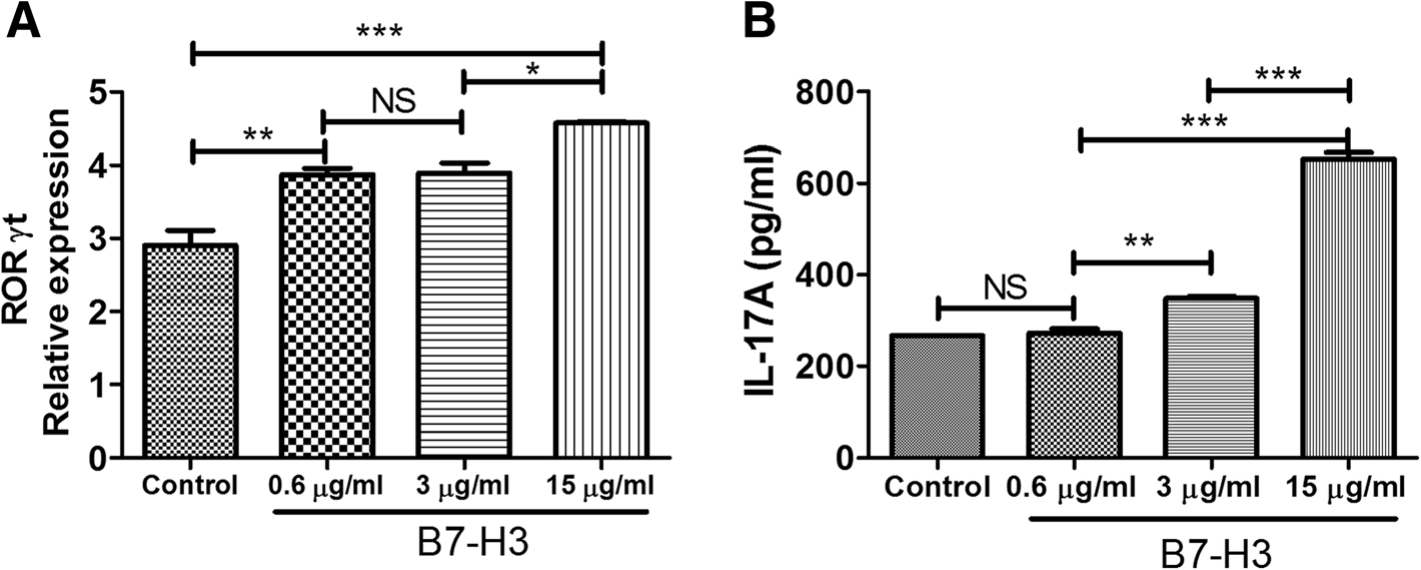
Function of B7-H3 on CD4^+^ T cells to induce expression of ROR-γt and IL-17A. Different concentrations of B7-H3 fusion protein (0.6 μg / ml, 3 μg / ml and 15 μg / ml) or human IgG as control group co-cultured with CD4^+^ T cells for 24 h. The expression of transcription factor ROR-γt (**a**) and IL-17A (**b**) was detected by real-time PCR and ELISA, respectively. P < 0.05 was considered statistically significant

Different concentrations of B7-H3 fusion protein (0.6 μg / ml, 3 μg / ml and 15 μg / ml) or human IgG as control group co-cultured with CD4^+^ T cells and separated using magnetic beads after 24 h. The expression of transcription factor ROR-γt was detected by real-time PCR, and the concentrations of IL-17A in supernatant was measured by ELISA. As shown in Fig. 3, high concentrations of B7-H3 (15μg/ml) could enhance ROR-γt expression and increase IL-17A in supernatant.

### B7-H3 is the target of miR-29c confirmed by luciferase reporter assay

**Fig. 4:**
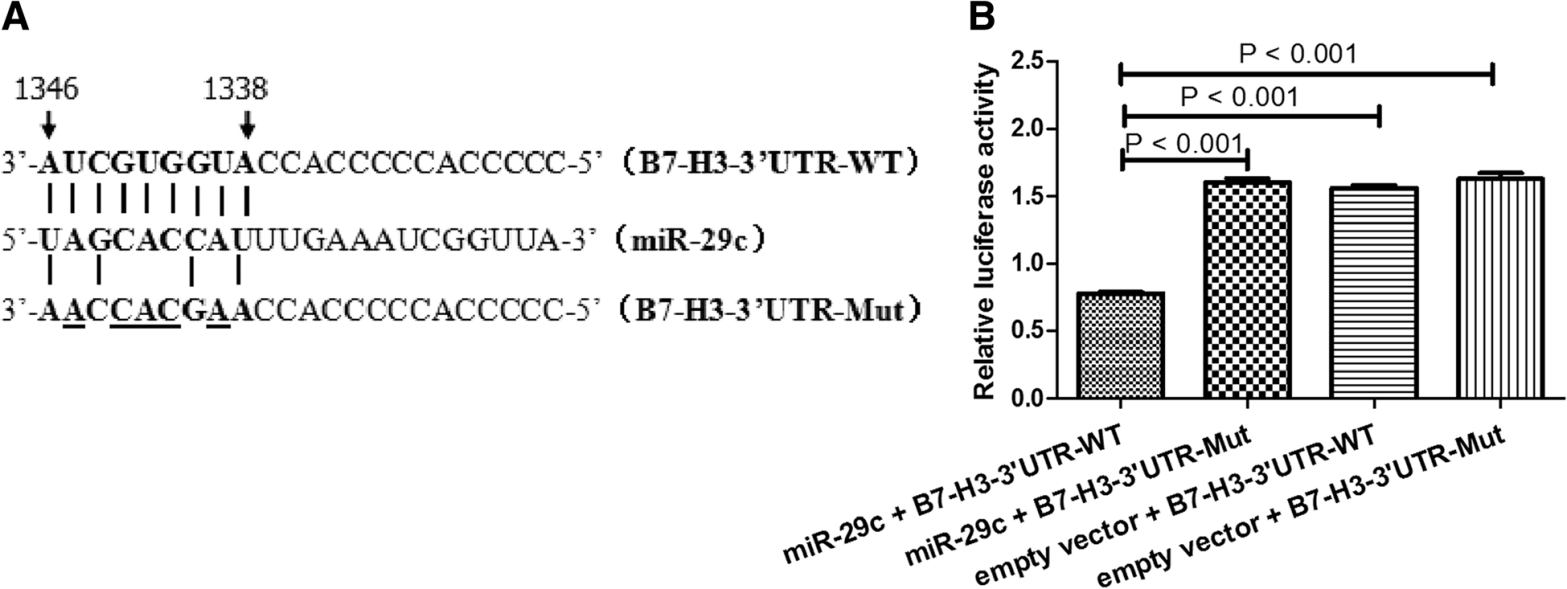
B7-H3 is the target of miR-29c confirmed by luciferase reporter assay. **a** The binding site of miR-29c in the 3’UTR of the B7-H3 mRNA and the mutant sequence of 3’UTR of B7-H3 mRNA. **b** Luciferase activity in THP-1 cells transiently cotransfected with pLenti-miR-29c or empty vector and B7-H3–3’UTR reporter plasmid or B7-H3–3’UTR-Mut reporter plasmid. Data are shown as mean ± SEM of 3 independent experiments

**Fig. 5:**
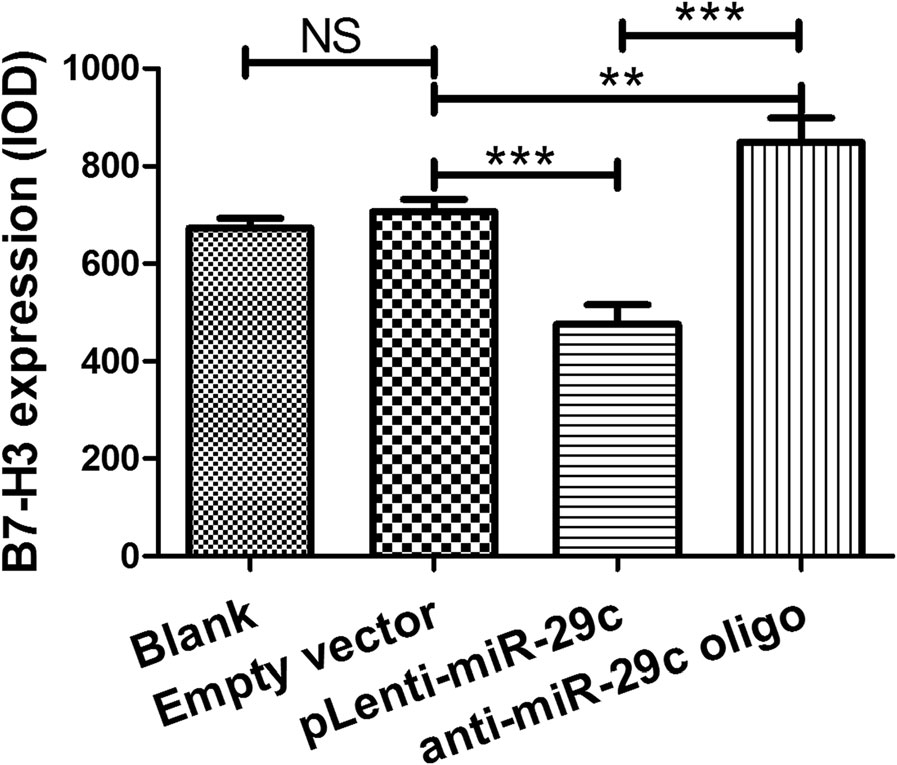
Transfection of miR-29c and B7-H3 detection by immunofluorescence staining. Comparison of grey levels of B7-H3 expression using Image-Pro Plus software. Data are presented as mean ± SEM of 3 independent experiments. ** *P* < 0.01; *** *P* < 0.001

To examine whether miR-29c actually could bind to the B7-H3 3′-UTR, we used a luciferase reporter construct (LightSwitch 3′-UTR reporter GoClone) with B7-H3 3′-UTR sequence using luciferase technology. The reporter was co-transfected into THP-1 cells with miR-29c and the luciferase activity was measured after 24 h. Transfection of miR-29c significantly inhibited the luciferase activity of the construct containing the B7-H3 3′-UTR compared to transfection of negative control miRNA or the construct containing the B7-H3 3′-UTR mutant, indicating that miR-29c directly bind to the B7-H3 3′-UTR as shown in Fig. 4. Meanwhile, overexpression or silencing of miR-29c could down or up-regulate the expression of B7-H3 in THP-1 cells using immunofluorescence assay (Fig. 5).

## Discussion

MPP is a common and frequently-occurring disease in children. MP was the most common pathogen of CAP in hospitalized children in Suzhou, China. In fact, MP infection in infants was even common [9–11]. In recent years, the incidence of SMPP shows a trend of increasing, and it has brought a heavy medical burden all over the world, especially in the Asian countries [11–14]. To our knowledge, the excessive host immunoreactions may play a part in the development of SMPP [14, 15]. This study aim to clarify the the possible mechanism of adaptive immune responses in MPP. In this study, we demonstrated that the costimulatory molecule B7-H3 had an important role in immune-inflammation reaction in MPP through promoting the polarization of Th17. Levels of sB7-H3 and IL-17 in supernatants were increased and miR-29c expression was decreased in childern with MPP, and there was a positive correlation between the expression of sB7-H3 and IL-17.

It was reported that higher frequencies of Th17 cells and higher levels of IL-17 were detected compared to healthy group, but there were no significant difference with the frequencies of Tregs and the levels of TGF-β1 in the patients with SMPP. It was indicated that Th17 cells played a vital role in SMPP [15, 16]. Both antigen or cleavage products of MP could induce high expression of IL-17A in bronchoalveolar lavage fluid (BALF) in mice [16, 17], and they could stimulate proliferation of mouse lymphocytes and then secrete IL-17A by Th17 cells [4]. Taken together, these studies suggested that Th17 cells and IL-17 played an important role in MPP, but the exact mechanism remains unclear. Several studies have shown that MP can stimulate alveolar macrophages produce IL-23, and then IL-23 induces IL-17 production in activated CD4^+^ T cells. In contrast, blocking of IL-23 alone resulted in a significant reduction of Mp-induced IL-17 in BALF of mice [17, 18]. While the Th17 cell differentiation was engaged by the IL-23 [18, 19],which demostrated the Th17 cell differentiation induced by MP was engaged by the IL-23.

It is now known that Th activation and differentiation require dual signal stimulation. Co-stimulatory molecules are a heterogenous group of cell surface molecules that act to amplify or counteract the initial activating signals provided to T cells from the T cell receptor (TCR) following its interaction with an antigen/major histocompatibility complex (MHC), thereby influencing T cell differentiation [19, 20]. Th activation and differentiation is associated with costimulatory molecules expressed on antigen presenting cell including B7 family molecules. Recently, Luo et al. evaluated the functions of B7-H3 in the regulation of Th1, Th2, and Th17 subsets in experimental autoimmune encephalomyelitis, experimental asthma, and collagen-induced arthritis using B7-H3 deficient mouse (B7-H3 KO), it suggested that B7-H3 has function on Th1/Th17 and could enhance IFN-γ and IL-17 production [20, 21]. This is consistent with our findings that B7-H3 plays an role in immunopathogenesis of children with MPP through regulating of Th17 differentiation and enhancing secretion IL-17. In present study, we also demonstrated that there was a positive correlation between the levels of sB7-H3 and IL-17 in children with MPP.

The gene expression of B7-H3 controlled by many factors including miRNAs. A previous study indicated that MicroRNA-187, down-regulated in clear cell renal cell carcinoma and associated with lower survival, inhibits cell growth and migration though targeting B7-H3 [21, 22]. There are 13 kinds of miRNAs that can target the regulation of B7-H3 in the breast cancer patients, and only miR-29c is associated with the prognosis of breast cancer patients [22, 23]. The aim of our study is to determine the level of sB7-H3 and IL-17 expression in children with MPP. The luciferase reporter assay were performed to confirm B7-H3 is the direct target of miR-29c. The gradual regulation in axis of miR-29c/B7-H3/Th17 is confirmed in vitro, and the expression of miR-29c, sB7-H3, IL-17 during the acute and convalescent phase is consistent with the result in vitro. The association between sB7-H3 and lactate dehydrogenase (LDH) was not statistically significant, but there was related trends. LDH has been confirmed to be associated with the severity of MPP, and LDH is an indicated marker for steroid therapy for MPP [23, 24]. However, there was no association between miR-29c and sB7-H3 in children with MPP, it is probably because there are other miRNAs to regulate B7-H3 expression except for miR-29c [21–26]. It is interesting that the serum level of miR-29c expression was negatively correlated with the *M. pneumoniae* specific IgG and IgM level during the acute phase, which suggested that children with low expression of miR-29c may have an excessive inflammatory immune response to MP infection. However the mechanism is require to explored.

## Conclusions

our study demonstrated that the axis of miR-29c/B7-H3/Th17 plays vital role in children with MPP through excessive inflammation. miR-29c and B7-H3 may be the new target for the prevention and treatment of MPP, and may be the novel and potential biomarkers for the assessment of prognosis.

## Abbreviations

ARDS: Acute respiratory distress syndrome
B7-H3: Co-stimulatory molecule B7-H3
BALF: Bronchoalveolar lavage fluid
CAP: Community-acquired pneumonia
ELISA: Enzyme-linked immunosorbent assays
M.Pneumoniae: Mycoplasma pneumoniae
miR-29c: MircoRNA-29c
MPP: *Mycoplasma pneumoniae* pneumonia
PCR: Real-time polymerase chain reaction
SMPP: Severe m*ycoplasma pneumoniae* pneumonia
